# BMP4 inhibits the proliferation of breast cancer cells and induces an MMP-dependent migratory phenotype in MDA-MB-231 cells in 3D environment

**DOI:** 10.1186/1471-2407-13-429

**Published:** 2013-09-22

**Authors:** Minna Ampuja, Riikka Jokimäki, Kati Juuti-Uusitalo, Alejandra Rodriguez-Martinez, Emma-Leena Alarmo, Anne Kallioniemi

**Affiliations:** 1 Institute of Biomedical Technology, University of Tampere and BioMediTech, Tampere, Finland; 2 Fimlab Laboratories, Tampere, Finland

**Keywords:** 3D culture, Matrigel, Breast cancer, BMP4, Proliferation, Migration

## Abstract

**Background:**

Bone morphogenetic protein 4 (BMP4) belongs to the transforming growth factor β (TGF-β) family of proteins. BMPs regulate cell proliferation, differentiation and motility, and have also been reported to be involved in cancer pathogenesis. We have previously shown that BMP4 reduces breast cancer cell proliferation through G1 cell cycle arrest and simultaneously induces migration in a subset of these cell lines. Here we examined the effects of BMP4 in a more physiological environment, in a 3D culture system.

**Methods:**

We used two different 3D culture systems; Matrigel, a basement membrane extract from mouse sarcoma cells, and a synthetic polyethylene glycol (PEG) gel. AlamarBlue reagent was used for cell proliferation measurements and immunofluorescence was used to determine cell polarity. Expression of cell cycle regulators was examined by Western blot and matrix metalloproteinase (MMP) expression by qRT-PCR.

**Results:**

The MCF-10A normal breast epithelial cells formed round acini with correct apicobasal localization of α6 integrin in Matrigel whereas irregular structures were seen in PEG gel. The two 3D matrices also supported dissimilar morphology for the breast cancer cells. In PEG gel, BMP4 inhibited the growth of MCF-10A and the three breast cancer cell lines examined, thus closely resembling the 2D culture conditions, but in Matrigel, no growth inhibition was observed in MDA-MB-231 and MDA-MB-361 cells. Furthermore, BMP4 induced the expression of the cell cycle inhibitor p21 both in 2D and 3D culture, thereby partly explaining the growth arrest. Interestingly, MDA-MB-231 cells formed large branching, stellate structures in response to BMP4 treatment in Matrigel, suggestive of increased cell migration or invasion. This effect was reversed by Batimastat, a broad-spectrum MMP inhibitor, and subsequent analyses showed BMP4 to induce the expression of *MMP3* and *MMP14*, that are thus likely to be responsible for the stellate phenotype.

**Conclusions:**

Taken together, our results show that Matrigel provides a more physiological environment for breast epithelial cells than PEG gel. Moreover, BMP4 partly recapitulates in 3D culture the growth suppressive abilities previously seen in 2D culture and induces an MMP-dependent migratory phenotype in MDA-MB-231 cells.

## Background

Bone morphogenetic protein 4 (BMP4) is a growth factor that belongs to the bone morphogenetic protein (BMP) family, which comprises the majority of the transforming growth factor β (TGF-β) –superfamily [1]. BMPs are extracellular ligands that bind serine/threonine receptors on the cell membrane and signal through intracellular SMAD mediators as well as through other pathways such as the MAP kinase pathway. BMPs were first found due to their bone-inducing effects and later studies showed them to be also powerful developmental regulators. For example, BMP4 is involved in gastrulation, mesoderm formation, hematopoiesis and the development of several organs and tissues including mammary gland [2-4].

Due to their multifunctionality, BMPs have been increasingly studied as potential players in cancer. BMP4 expression in cancer varies and both increased and decreased expression has been reported depending on the tissue of origin [5]. In breast cancer, strong *BMP4* expression has been found in both cell lines and tissues [6-8] and immunohistochemical data indicate that BMP4 protein is expressed in one fourth to half of primary tumors [9]. Functional studies in multiple malignancies suggest that BMP4 typically causes reduced growth and increased migration of cancer cells [5]. We have previously shown, using a large set of breast cancer cell lines, that BMP4 treatment systematically inhibits proliferation in all cell lines and simultaneously increases migration of MDA-MB-231, MDA-MB-361 and HCC1954 cells, but reduces migrativeness of T-47D cells [10]. Similarly, Guo and colleagues [6] demonstrated increased migration and decreased proliferation upon BMP4 overexpression in MDA-MB-231 and MCF-7 breast cancer cells. These data were corroborated by an *in vivo* study where inhibition of BMP4 signaling decreased metastasis of MDA-MB-231 breast cancer cells [11]. Yet there is one study where BMP4 reduced migration of MDA-MB-231 cells [12]. Nevertheless, the majority of the data implies that BMP4 has a dualist effect on breast cancer cells, with inhibition of cell proliferation and induction of a migratory phenotype.

The aforementioned *in vitro* functional studies were done using cells growing as two-dimensional (2D) monolayer. However, there is an increasing interest in culturing cells in a more biologically relevant three-dimensional (3D) environment [13]. This has been generally achieved by growing cells in synthetic scaffolds or gels of biological or synthetic origin [14]. Matrigel, basement membrane extract from mouse sarcoma, is the most commonly used biological scaffold and consists mainly of laminin, collagen IV and various growth factors [15]. Other biological materials that are often used include collagen, alginate and hyaluronic acid [14]. Synthetic gels have been developed as alternatives to the biological gels due to the difficulties in defining the exact composition of the biological materials and the fact that they may suffer from batch-to-batch variability [14]. Synthetic gels, mainly different polymers, such as polyethylene glycol and polyvinyl alcohol, have a constant composition and are easy to manipulate. However, they may not adequately represent the complicated extracellular matrix (ECM) that surrounds cells in tissues [14,16].

Various cell types, including epithelial, neural and endothelial cells, have been successfully grown in 3D and are capable of forming structures that resemble the normal tissue organization [15]. For example, normal immortalized mammary epithelial cells, such as the MCF-10A cells, form polarized acini structures in Matrigel, reminiscent of the normal breast architecture [17], whereas breast cancer cells generate more variable structures [18]. Similarly, biologically appropriate cellular organization has been observed e.g. for epithelial and neural cells in different synthetic gels [19-21]. More importantly, the shift from 2D to 3D culture also results in changes in gene expression in multiple tissue types [13,22-25]. For example, breast epithelial cells begin to produce milk proteins when grown in Matrigel [25].

Previous data from us and others showed that BMP4 is able to reduce the growth of breast cancer cells whilst inducing cell migration and invasion [6,10,11]. Here we utilized two different 3D culture systems to evaluate whether these phenotypes persist under more physiological culture conditions and further explored the mechanisms of BMP4-induced changes in cell proliferation and mobility.

## Methods

### Cell lines

The MCF-10A, MDA-MB-231, MDA-MB-361, BT-474 and T-47D cell lines were purchased from ATCC (Manassas, VA, USA) and cultured according to ATCC instructions, except for MCF-10A, which was maintained as previously described [17]. In 3D experiments, MDA-MB-231 and MDA-MB-361 cells were cultured in DMEM (Sigma-Aldrich, St. Louis, MO, USA). For MCF-10A cells a reduced concentration of EGF (5 ng/ml) was used in Matrigel [17].

### BMP4 and inhibitor treatments

rhBMP4 (100 ng/ml, R&D Systems, Minneapolis, MN, USA), BMP antagonist Gremlin (1 μg/ml, R&D Systems), MMP inhibitor Batimastat (10 μM, Millipore, Billerica, MA, USA) or a combination of these was added to the medium at the start of the experiments and replenished every two to three days as the medium was exchanged. Vehicle-treated cells received BMP4 dilution buffer (4 mM HCl with 0.1% BSA), Gremlin dilution buffer (0.1% BSA in PBS), Batimastat dilution buffer (DMSO), or a combination of these. All experiments were done in two to six replicates and were repeated at least twice.

### Cell proliferation assay

Medium with 10% alamarBlue (Invitrogen) was added to the cells and incubated for 1 hour (2D culture) or 4 hours (Matrigel and PEG gel). Medium was collected and fluorescence (excitation wavelength 560 nm, emission wavelength 590 nm) measured using Tecan infinite F200 Pro plate reader (Tecan, Männedorf, Switzerland). Additionally, the number of cells in 2D culture was counted using the Z1 Coulter Counter (Beckman Coulter, Fullerton, CA) at indicated time points. The experiments were done in four to six replicates and repeated at least twice.

### Cell cycle

MCF-10A cells were cultured on 24-well plates and analyzed 3 and 5 days after first addition of BMP4. The cells were stained with PI as described [26]. The cell cycle distribution was determined using the Accuri C6 flow cytometer (Accuri, Ann Arbor, MI, USA) and ModFit LT 3.0 (Verity software house, USA). The experiment was performed twice with six replicates.

### 3D Matrigel assay

Cells were cultured on growth factor-reduced Matrigel (BD Biosciences, Franklin Lakes, NJ, USA) using the overlay method [17]. Briefly, 4-chambered Lab-Tek chamber slides (Nalge Nunc International, Rochester, NY, USA) or 24-well plates were coated with Matrigel. Cells (2.0 × 10^4^ cells/ml for MDA-MB-231 and T-47D, 2.4 x 10^4^ cells/ml for MCF-10A, 6.0 × 10^4^ cells/ml for BT-474 and 1.2 × 10^5^ cells/ml for MDA-MB-361) suspended in 2.5% Matrigel solution were added on coated chamber slides and allowed to grow up to 17 days.

### 3D PEG gel assay

MMP-degradable polyethylene glycol (PEG) gel with RGD peptides was purchased from QGel (Lausanne, Switzerland). Briefly, 400 μl of Buffer A was mixed with QGelTM MT 3D Matrix powder, before addition of 100 μl of cell suspension (given a final concentration of 1.4 × 10^5^ cells/ml for MCF-10A, 1.0 × 10^5^ cells/ml for MDA-MB-231, 8.0 × 10^4^ cells/ml for T-47D, and 4.0 × 10^5^ cells/ml for MDA-MB-361). Drops of 40 μl were applied into a disc caster and after 30 min incubation at 37°C the gelled discs were removed and placed on 24-well plates with 1 ml of medium per well. The cells were allowed to grow up to 18 days.

### Immunofluorescence

The MCF-10A cells in Matrigel and PEG gel were fixed in 4% paraformaldehyde for 1 hour at 37°C followed by permeabilization with 0.1% Triton-X100 for 45 min at room temperature and blocking with 3% BSA for 1.5 hours at 37°C. The fixed cells were incubated with mouse monoclonal anti-α6 integrin antibody (1:300, Abcam, Cambridge, UK) for 1.5 hours at 37°C. The secondary goat anti-mouse Alexa Fluor 488 (1:200, Invitrogen) was used similarly. The cells were stained with DAPI (Invitrogen) and mounted with Vectashield (Vector Laboratories, Burlingame, CA, USA). Images were taken with Zeiss Axio Imager. M2 microscope (Carl Zeiss, Oberkochen, Germany) connected to an ApoTome slider module (Carl Zeiss).

### Image analysis

Images were taken from the cells in Matrigel and PEG gel using Olympus IX71 microscope (Olympus, Tokyo, Japan) and processed with ImageJ (U.S. National Institutes of Health, Bethesda, MD, USA). Four images from each experiment at designated time points were analyzed and the average area covered by the cells was calculated.

### Protein extraction

The cells were collected 24 hours or 5 days (2D culture) and 4 or 7 days (Matrigel) after first addition of BMP4. Matrigel was first dissolved by adding cold PBS with 5 mM EDTA and the cells were kept on ice for 15 min. The cell-Matrigel solution was then collected, kept on ice for 30 min and centrifuged for 15 min at 3300 × g, at 4°C. Cells were lysed and protein concentration measured as previously described [10].

### Western blot

Fifty μg of protein was loaded onto SDS-PAGE gels. After gel electrophoresis, the proteins were transferred to a PVDF membrane. The following primary antibodies (Santa Cruz Biotechnology, CA, USA) and dilutions were used: p21 (1:100), Cdk4 (1:1000), Cdc2 (1:1000), p-Cdc2 (Thr14/Tyr15, 1:200), p27 (1:500), p16 (1:100), p15 (1:200), Cyclin B1 (1:200), Cyclin B2 (1:100) and Cyclin D1 (1:200). All antibodies were rabbit polyclonal, with the exception of p16 (mouse monoclonal) and Cyclin B2 (goat polyclonal). In addition, a mouse monoclonal anti-GTF2H1 antibody (1:1000, Abcam) was used. Proteins were detected using the BM Chemiluminescence Western Blotting kit (Roche, Mannheim, Germany) according to manufacturer’s instructions. Anti-mouse/rabbit secondary antibody (1:5000, Roche) was used for all antibodies, except for Cyclin B2, which was detected with anti-goat secondary antibody (1:5000, Santa-Cruz Biotechnology). The membranes were stripped and probed with β-tubulin (Sigma-Aldrich) as a loading control.

### Quantitative RT-PCR

The expression of *MMP-1*, -*2*, -*3*, -*7*, -*9*, -*14* and *ADAM17* was examined in BMP4- and vehicle-treated MDA-MB-231 and BT-474 (*MMP3* and *MMP14* only) cells grown for 14 days in Matrigel. The cells were harvested as described above for protein extraction. Total RNA was extracted using RNeasy Mini kit (Qiagen, Valencia, CA) and was reverse transcribed using SuperScriptTM III First-Strand Synthesis System for RT-PCR (Invitrogen) as described [7]. qRT-PCR was performed using gene specific primers and UPL probes (Roche, Additional file 1: Table S1) and the LightCycler equipment (Roche) as described [27] with 1.2 μM concentration of primers and probes and the following program: 10 min denaturation at 95°C followed by 45 cycles of 10 s denaturation at 95°C, 10 s annealing at 55°C and 15 s elongation at 72°C. The experiments were done in three replicates and the expression levels were normalized using Phosphoglycerate kinase 1 (*PGK1*) housekeeping gene.

### Statistical analyses

The difference between BMP4- and vehicle-treated samples in cell proliferation and area analysis was evaluated using the Mann–Whitney test with GraphPad Prism 4 (GraphPad Software, La Jolla, CA, USA). A P-value of less than 0.05 was considered significant.

## Results

### BMP4 inhibits the growth of MCF-10A cells in both 2D and 3D cell culture

**Figure 1:**
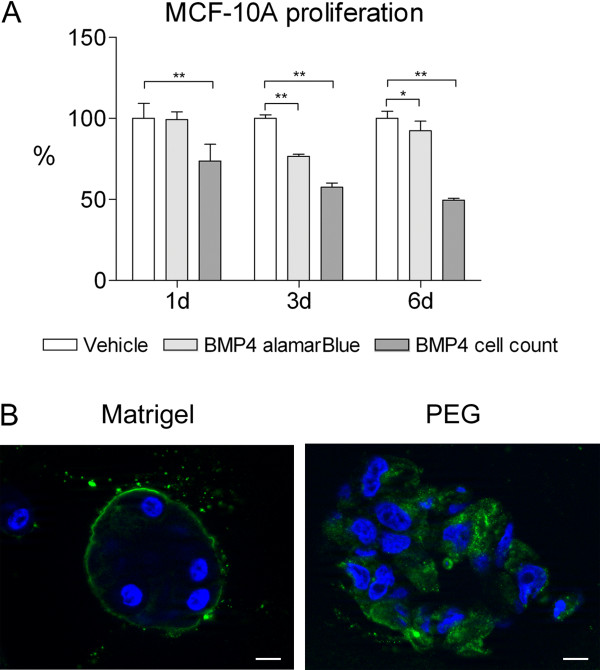
**Characterization of MCF-10A cells in 2D and 3D culture. (A)** BMP4 treatment significantly reduces the proliferation of MFC-10A cells in 2D culture. Cells were grown in the presence of 100 ng/ml BMP4 or vehicle and proliferation was measured using the alamarBlue reagent and by counting the cells at indicated time points. Relative proliferation (mean + s.d.) compared to vehicle is shown. *P < 0.05, **P < 0.01. **(B)** MCF-10A cells form polarized structures in Matrigel but not in PEG gel. The cells were grown in Matrigel for 14 and in PEG gel for 11 days, fixed, and immunofluorescently labeled with polarization marker α6-integrin antibody (green). The nuclei were stained with DAPI (blue). Images were taken with Zeiss Axio Imager.M2 microscope. Scale bar 10 μm.

We began the study using an immortalized breast epithelial cell line MCF-10A, which is widely used in 3D cultures. However, since no previous data existed, we first tested the effects of BMP4 on these cells in standard 2D culture. Similar to breast cancer cell lines [10], BMP4 decreased the proliferation of the MCF-10A cells as determined by cell counting and alamarBlue (Figure 1A). A highly significant decrease in cell number was evident at day 3 and day 6 (42% and 50%, respectively, as compared to vehicle; P < 0.01).

In 3D assays, both biological (Matrigel) and synthetic (polyethylene glycol, PEG gel) materials were used. In Matrigel, MCF-10A cells formed round acini-like structures with correct apicobasal polarity of the acini, as illustrated by the basal localization of α6-integrin (Figure 1B, left panel). In contrast, MCF-10A cells grown in PEG gel demonstrated a disordered structure with no obvious lumen formation and no basal localization of α6-integrin (Figure 1B, right panel).

**Figure 2:**
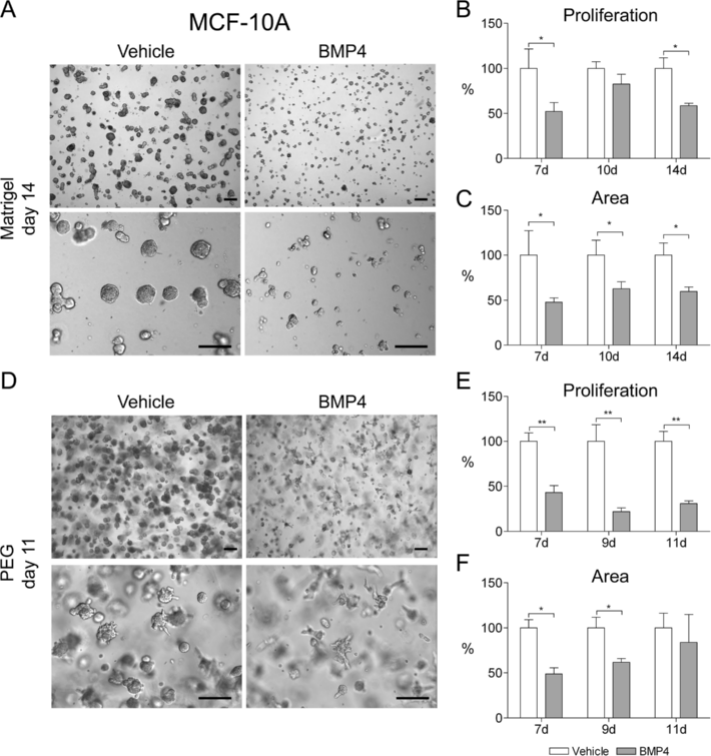
**BMP4 inhibits MCF-10A cell growth in 3D cell culture.** Cells were grown in Matrigel **(A-C)** or in PEG gel **(D-F)** supplemented with 100 ng/ml BMP4 or vehicle. Images were captured with Olympus IX71 microscope and representative examples from day 14 (Matrigel, panel **A**) and day 11 (PEG gel, panel **D**) are shown. Scale bars 200 μm. (b, e) Cell proliferation was measured using the alamarBlue reagent at indicated time points and relative proliferation (mean + s.d.) compared to vehicle is presented. **(C, F)** The area covered by cell clusters was measured from images taken at indicated time points using ImageJ and the relative mean area and s.d. compared to vehicle is shown. *P < 0.05, **P < 0.01.

When MCF-10A cells in Matrigel were treated with BMP4 (100 ng/ml), there was no change in the acinar morphology but proliferation of the cells was reduced (Figure 2A-C). The proliferation rate (as measured by alamarBlue) was decreased by 41% at day 14 in BMP4-treated cells as compared to vehicle-treated cells (P < 0.05, Figure 2B). Accordingly, BMP4 also significantly decreased the size of the acini structures as evidenced by a 40% reduction in the total area covered by the cell clusters at day 14 (P < 0.05, Figure 2C).

In PEG gel, vehicle-treated MCF-10A cells mainly formed round cell clusters with occasional protrusions whereas BMP4-treated cells formed irregularly shaped elongated structures with high numbers of protrusions (Figure 2D). In addition, BMP4 inhibited the proliferation of the MCF-10A cells by 69% at day 11 as compared to the vehicle (P < 0.005, Figure 2E). Analysis of the area covered by cells revealed a maximum reduction of 51% at day 7 after BMP4 treatment (P < 0.05, Figure 2F).

### BMP4 induces different phenotypes in breast cancer cells in 3D

**Figure 3:**
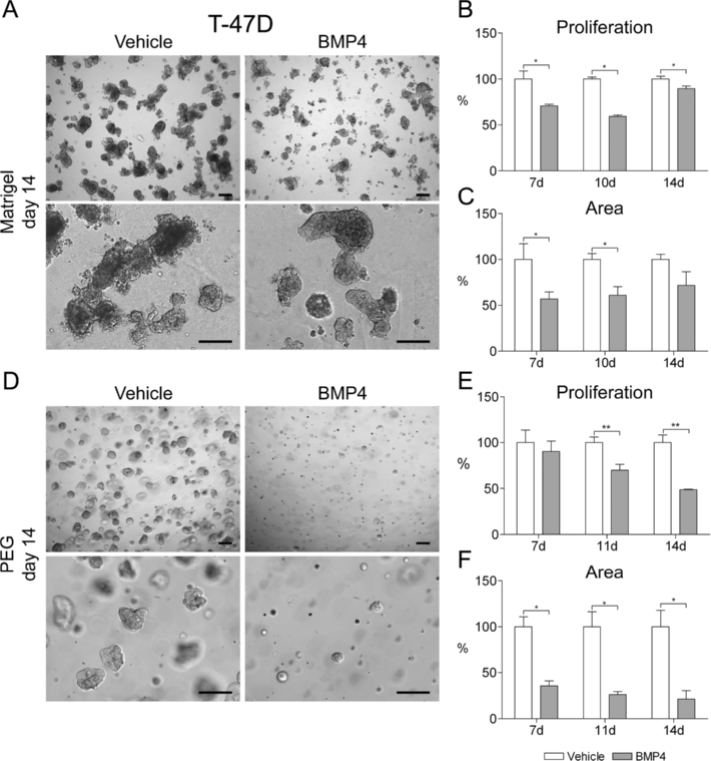
**BMP4 inhibits T-47D cell growth in 3D cell culture.** Cells were grown in Matrigel **(A-C)** or in PEG gel **(D-F)** and supplemented with 100 ng/ml BMP4 or vehicle. Images were taken as indicated in Figure 2 and representative examples from day 14 are shown. Scale bars 200 μm. **(B, E)** Cell proliferation and **(C, F)** area covered by cell clusters were measured and are presented as in Figure 2, *P < 0.05, **P < 0.01.

Next we examined the effects of BMP4 in 3D cultures of four breast cancer cell lines. The cell lines were chosen based on our previous data showing a prominent phenotype upon BMP4 stimulation in 2D; either G1 cell cycle arrest and growth inhibition (T-47D, BT-474, MDA-MB-361) and/or increased migration (MDA-MB-231, MDA-MB-361) [10, unpublished]. T-47D cells formed irregular raft-like structures in Matrigel (Figure 3A). BMP4 treatment did not induce any obvious changes in the morphology of the cell clusters but inhibited cell proliferation (29% at day 7, 41% at day 10 and 10% at day 14 as compared to vehicle, P < 0.05, Figure 3A-B). The size of the area covered by cells was similarly reduced by 43% and 39% at days 7 and 10, respectively (P < 0.05, Figure 3C). At day 14 the difference was 28% but just failed to reach statistical significance (Figure 3C). In PEG gel, the T-47D cell structures were either round or polygonal in shape, in both BMP4- and vehicle-treated samples (Figure 3D). BMP4 induced a distinct decrease in cell proliferation at days 11 and 14 (30% and 51%, respectively, as compared to vehicle, P < 0.01, Figure 3E). Consequently, there was a significant reduction in the size of the cell area, ranging from 64% at day 7 to 79% at day 14 (P < 0.05, Figure 3F).

**Figure 4:**
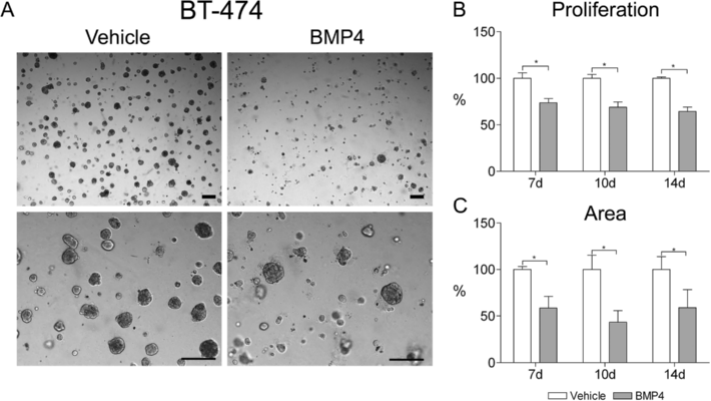
**BMP4 inhibits BT-474 cell growth in 3D cell culture. (A)** Cells were grown in Matrigel and supplemented with 100 ng/ml BMP4 or vehicle. Images were taken as indicated in Figure 2 and representative examples from day 14 are shown. Scale bars 200 μm. **(B)** Cell proliferation and **(C)** area covered by cell clusters were measured and are presented as in Figure 2, *P < 0.05.

For BT-474 cells, the consequences of BMP4 treatment were first examined in 2D culture due to lack of previous information. A significant decrease in cell count was detected in BMP4-treated cells as compared to vehicle (30% at day 3 and 70% at day 6, P < 0.01, Additional file 2: Figure S1). In Matrigel the cells formed dense, mostly round structures (Figure 4A). Proliferation was reduced by 26% already at day 7 and continued to decrease up to 36% at day 14 after BMP4-treatment (P < 0.05, Figure 4B). A concomitant reduction of 40% to 50% on average could be seen in the area measurements (P < 0.05, Figure 4C).

MDA-MB-361 cells grew very slowly in both 3D environments and therefore were allowed to grow up to 18 days (Additional file 3: Figure S2). In Matrigel, the cells formed small mostly round masses, and BMP4 treatment induced no consistent changes in proliferation, area or morphology of the cells (Additional file 3: Figure S2A-C). In contrast, in PEG gel BMP4 significantly decreased proliferation at day 11 through day 18 (15% and 28%, respectively, as compared to vehicle, P < 0.01, Additional file 3: Figure S2E). In addition, BMP4 decreased the size of the area covered by cells, with a maximum reduction of 48% at day 11 (P < 0.05, Additional file 3: Figure S2F). However, no changes in the morphology of the cell structures were observed in PEG gel with both BMP4 and vehicle treatments resulting in round cell clusters.

**Figure 5:**
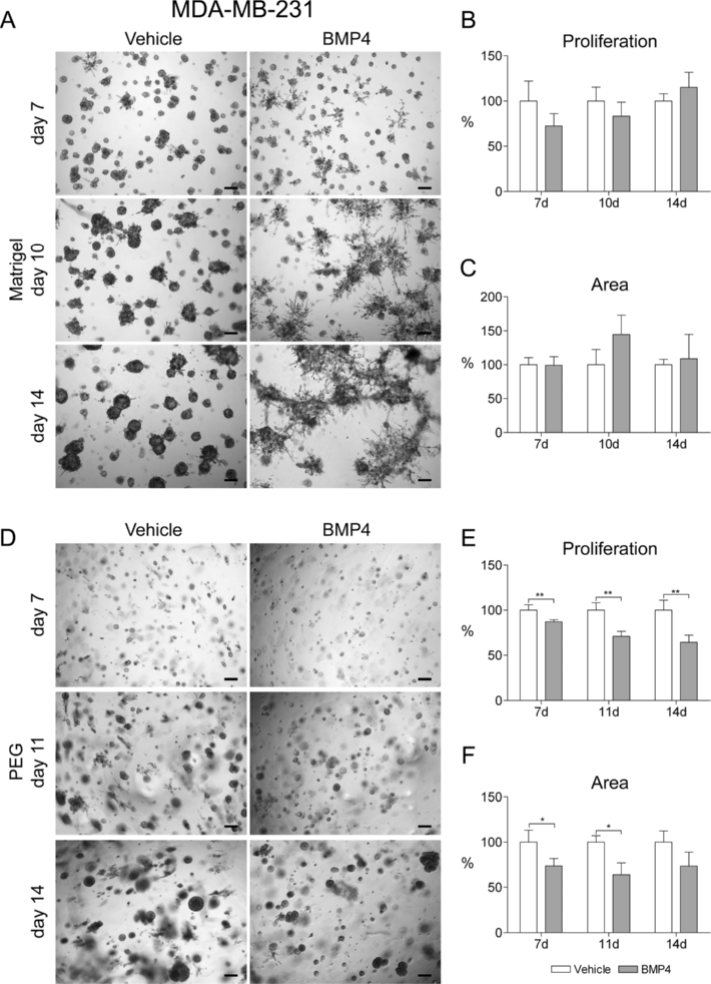
**BMP4 induces a stellate phenotype and reduces the growth of the MDA-MB-231 cells in 3D cell culture.** Cells were grown in Matrigel **(A-C)** or in PEG gel **(D-F)** supplemented with 100 ng/ml BMP4 or vehicle. Images were taken as indicated in Figure 2 and representative examples from days 7, 10 and 14 for Matrigel and days 7, 11 and 14 for PEG gel are shown. Scale bars 200 μm. **(B, E)** Cell proliferation and **(C, F)** area covered by cell clusters were measured and are presented as in Figure 2, *P < 0.05, **P < 0.01.

MDA-MB-231 cells formed mostly dense and compact round or oval structures in Matrigel with occasional branches (Figure 5A). Interestingly, BMP4 had a major impact on the morphology of the cells. It induced the formation of large branching stellate structures, which extended over large areas of the gel (Figure 5A). The first evidence on this effect was seen already at day 7, but it became prominent after 10 days in culture (Figure 5A). On the other hand, BMP4 did not have an effect on the proliferation of the MDA-MB-231 cells as measured by alamarBlue or the area covered by the cells (Figure 5B and 5C). It should be noted that the latter result is hindered by the difficulties in accurately measuring the area of the BMP4-induced stellate structures. In PEG gel, no branching was observed and the MDA-MB-231 cell masses were typically round or irregularly shaped in both BMP4- and vehicle-treated samples (Figure 5D). Interestingly, BMP4 significantly inhibited proliferation of the MDA-MB-231 cells in PEG gel, with a 36% reduction by day 14 (P < 0.01, Figure 5E). Similarly, the area covered by the cells was diminished by a maximum of 36% at day 11 (P < 0.05, Figure 5F).

### BMP4-induced growth arrest is partly explained by induction of p21 expression

**Figure 6:**
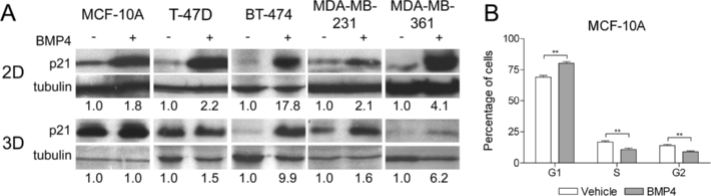
**The expression of cell cycle inhibitor p21 is altered by BMP4. (A)** MCF-10A cells were treated with 100 ng/ml BMP4 (+) or vehicle (−) for 5 days and the cancer cell lines for 24 hours when grown as monolayers (2D). In Matrigel (3D), the cells were grown and treated for 4 (MDA-MB-361) or 7 days. The expression of p21 was analyzed by western blot. Tubulin was used as a loading control and relative expression levels were calculated with ImageJ. **(B)** BMP4 treatment leads to G1 arrest of MCF-10A cells. The cell cycle was determined by flow cytometry at day 5 after the beginning of the treatments. The fraction (mean + s.d.) of cells in phases G1, S and G2 are shown. **P < 0.01.

We have previously shown that the growth inhibition caused by BMP4 in breast cancer cell lines growing in monolayer culture is due to a G1 cell cycle arrest [10]. To investigate this further, the effect of BMP4 on the expression of 11 known cell cycle regulators was measured in T-47D and MDA-MB-361 cells grown for 24 hours in 2D. A change in the expression of the cell cycle inhibitor p21, phosphorylated CDC2 and Cyclins B1 and B2 was seen in both cell lines, with at least a 2-fold difference in one of the cell lines (Additional file 4: Figure S3). Among these, induction of p21 was the most prominent (4.1-fold in MDA-MB-361 and 2.2-fold in T-47D) and was thus selected for further evaluation. We verified that p21 expression was also induced by BMP4 in 2D culture of MDA-MB-231 and BT-474 cells (Figure 6A). In MCF-10A cells, distinct p21 induction (1.8-fold) was evident only after a prolonged (5 days) BMP4 treatment (Figure 6A) and was accompanied by a G1 cell cycle arrest (G1 phase fraction 80% vs. 69% in BMP4- and vehicle-treated cells, respectively, P < 0.05, Figure 6B). In Matrigel, the p21 levels were determined at day 4 or 7 after BMP4 treatment. BMP4 had no effect on p21 expression in MCF-10A cells whereas it did induce p21 expression in T-47D, BT-474, MDA-MB-361 and MDA-MB-231 cells (Figure 6A).

### Induction of a stellate phenotype in MDA-MB-231 cells is MMP-dependent

**Figure 7:**
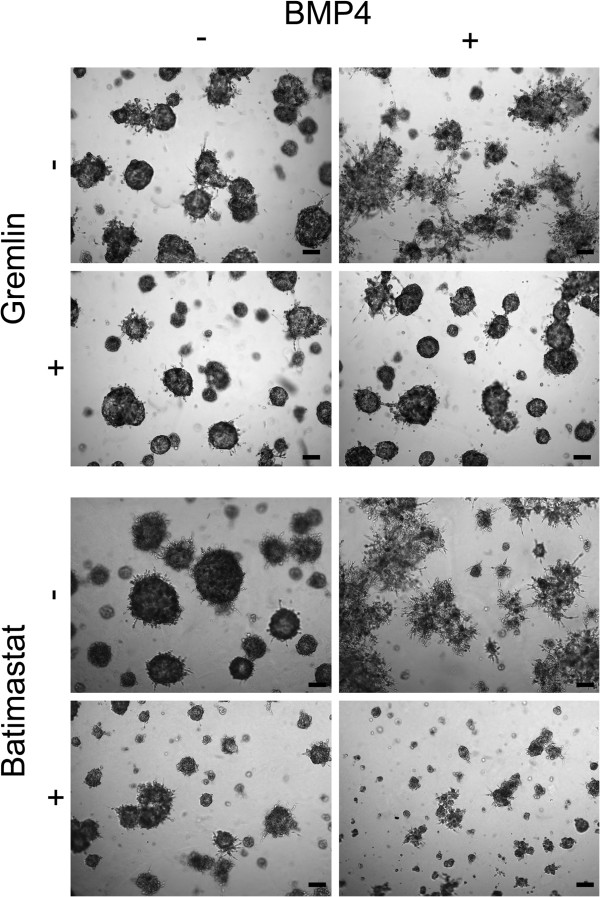
**BMP4 antagonist Gremlin and MMP inhibitor Batimastat reverse the stellate phenotype of MDA-MB-231 cell clusters in Matrigel.** The cells received 1 μg/ml Gremlin, 10 μM Batimastat and/or 100 ng/ml BMP4. Vehicle-treated cells were used as a control. Images were taken as indicated in Figure 2 and representative examples from day 14 are shown. Scale bar 200 μm.

To confirm that the stellate phenotype induced in the MDA-MB-231 cells in Matrigel was indeed dependent on BMP4, the cells were treated with BMP4 together with a BMP antagonist Gremlin, which inhibits the actions of BMP2, -4 and −7 [28]. Gremlin (1 μg/ml) alone had no effect on the morphology of the cells (Figure 7A). The cells treated with both Gremlin and BMP4 had similar morphology than vehicle-treated cells and thus Gremlin was able to reverse the stellate phenotype (Figure 7A).

We then speculated that the stellate phenotype may require the action of matrix metalloproteinases (MMPs). A broad-spectrum MMP inhibitor Batimastat was employed to test its potential in inhibiting the BMP4-induced phenotype. Batimastat (10 μM) alone resulted in a moderate reduction of growth of the cells as compared to vehicle-treated cells (Figure 7B). However, Batimastat was able to inhibit the formation of BMP4-induced stellate structures and, somewhat surprisingly, the combination of Batimastat and BMP4 resulted in a pronounced reduction in the size of the cell structures (Figure 7B).

As the stellate phenotype was reversed by an MMP inhibitor, we next examined the contribution of individual MMPs to this phenotype. Using quantitative RT-PCR, the expression levels of seven *MMP*s known to be targeted by Batimastat were measured in BMP4- and vehicle-treated MDA-MB-231 cells grown in Matrigel for 14 days. *MMP2*, *MMP7* and *MMP9* were not expressed in the MDA-MB-231 cells at a sufficient level to allow accurate measurements and there was no difference in *ADAM17* expression between BMP4-and vehicle-treated cells (data not shown). In contrast, there was a dramatic 19-fold increase in *MMP3* expression (P < 0.005) and a 3.7-fold increase in *MMP14* expression (P < 0.05) in BMP4-treated cells as compared to vehicle-treated cells. In addition, *MMP1* expression was 4.3 times higher in BMP4-treated cells but the difference was not statistically significant. To further verify that the induction of *MMP3* and *MMP14* was exclusively related to the BMP4-induced stellate phenotype in MDA-MB-231 cells, we measured *MMP3* and *MMP14* mRNA levels in one of the non-stellate cell lines, BT-474, under similar conditions and found that in this case BMP4 did not induce the expression of these *MMP*s (data not shown).

## Discussion

We have previously shown that BMP4 reduces proliferation and increases migration of breast cancer cells *in vitro*[10]. As these results were derived from cells grown in 2D monolayer culture, we set out to analyze the effect of BMP4 in a more physiological setting by employing 3D culture systems. We approached this issue by using both a biological gel (Matrigel, the standard 3D culture environment) and a synthetic material with RGD peptides and MMP-degradable peptide links (PEG gel).

The two materials studied provided dissimilar 3D environments as first evidenced by differences in the morphology of the normal and cancer cell clusters. The MCF-10A normal mammary epithelial cells had a polarized acini structure in Matrigel, as previously shown [17], while in PEG gel the cells formed irregular non-polarized structures. Similarly, the morphology of the different cancer cells varied between the two 3D models, with the structures formed in Matrigel again corresponding to those previously reported [18]. On a functional level, the growth response of cells to BMP4 treatment in PEG gel mirrored the 2D data, whereas in Matrigel more diverse effects were observed. These data could be explained by several factors. Matrigel contains multiple biologically active molecules, such as laminin, collagen IV and many growth factors [15], that are likely to impact the results obtained. Of these biologically active molecules, e.g. laminin-1 has been shown to be essential for correct polarization of primary luminal epithelial cells in collagen gels [29]. It has also been reported that 50 mM RGD peptide is an optimal concentration for acinar growth of MCF-10A cells in polyethylene glycol tetravinyl sulfone (PEG-VS) gel [30]. A lower concentration of RGD (50 μM) was present in the PEG gel used here, possibly explaining the lack of acinar formation. In addition, the stiffness and elasticity of the matrix is known to influence the cellular phenotype, including proliferation, differentiation and migration, in 3D environments [31-33]. To summarize, the differences in cell morphology and BMP4 response between the two materials tested demonstrate that the mere 3D architecture is not sufficient to mimic the biological effects of tissue environment. Based on the morphological characteristics, Matrigel seems to provide a more appropriate milieu for breast epithelial cells. While many synthetic 3D materials are entering the market, they should be used cautiously until their biological properties have been explored.

Previous data from us and others [6,10] clearly demonstrate that BMP4 reduces the proliferation of breast cancer cells in 2D culture, and similar results have been reported in other tumor types [5,34-37]. Here we extend these findings and first show the same growth suppressive effect of BMP4 in MCF-10A normal immortalized breast epithelial cells both in 2D and 3D environment. The 3D data from the breast cancer cell lines were more diverse. In PEG gel, BMP4 administration led to reduced cell proliferation for all cell lines tested, whereas in Matrigel two out of four cell lines (MDA-MB-231 and MDA-MB-361) did not display growth inhibition upon BMP4 treatment. In the case of MDA-MB-361, the very slow growth rate of the cells in 3D may have contributed to these findings, although the difference between responses in PEG gel and Matrigel implies an actual effect triggered by the different environments. Furthermore, the growth suppressive action of BMP4 seen in MDA-MB-231 cells in 2D [10] disappeared in 3D Matrigel and was overcome by a migratory phenotype. The response of the cells to biological molecules is known to change drastically in 3D, for example, many anticancer drugs are less effective in 3D culture [38]. Our data now suggest that the ability of BMP4 to reduce cell growth in 3D strongly depends on the material used. Nevertheless, cell line specific differences also exist and further highlight the importance of testing the impact of biological factors, including BMP4, in a proper environment.

BMP4 has been reported to induce G1 cell cycle arrest in cancer cells [10,39-41]. We now show for the first time that the mechanism behind this cell cycle arrest in breast cancer cells is the increased expression of the cell cycle inhibitor p21. This result is in concordance with previous reports in 2D culture of various normal and neoplastic cells [41-45]. Additionally, BMP2 has been shown to induce p21 expression in breast cancer cells [39,40,46]. Interestingly, BMP4 induced p21 expression in MDA-MB-231 and MDA-MB-361 cells in 3D even in the absence of growth inhibition, suggesting that p21 alone is not sufficient to induce growth arrest in these cells in 3D. Furthermore in MCF-10A cells, p21 induction and G1 cell cycle arrest were not evident until day 5 in 2D culture, even though a significant growth reduction was seen already at day 3. Likewise, in MCF-10A 3D culture no p21 induction was observed even after 7 days of BMP4 treatment. Therefore it seems likely that other factors are involved in the BMP4-mediated growth regulation in MCF-10A cells. Examination of a panel of cell cycle regulators in T-47D and MDA-MB-361 cells in 2D showed that BMP4 influenced the expression of multiple cell cycle proteins, including pCDC2, Cyclin B1 and Cyclin B2. These or other cell cycle regulators could thus contribute to the observed growth inhibition in MCF-10A cells as well. Previous studies have reported dysregulation of several cell cycle associated proteins, including Cyclin B1, CDC2, Rb, and E2F, after different stimuli in MCF-10A cells [47,48], emphasizing the fact that multiple factors may be simultaneously involved. Further research is needed to identify the specific cell cycle regulators influenced by BMP4 treatment in MCF-10A cells.

In most cases, BMP4 had no effect on the morphology of the cells grown in 3D environment, with the exception of MDA-MB-231 cells and MCF-10A cells. In PEG gel, MCF-10A cells formed irregular structures with small protrusions, the number of which increased upon BMP4 stimulation, indicating increased migration and/or invasion. This is consistent with previous results showing BMP4-induced invasive properties in mouse mammary epithelial cells in collagen gels [49]. In Matrigel, MDA-MB-231 cells formed stellate, branching structures in response to BMP4, which is in concert with previous observations of increased migration and invasion in 2D experiments [6,10]. Such structures were not observed in PEG gel, highlighting again the variation between the different 3D materials.

The MDA-MB-231 cells are known to be triple negative and represent the so-called basal subtype, whereas the remaining breast cancer cell lines used in this study are of luminal type [50]. We thus speculated whether the molecular subtype could explain the migratory response to BMP4 treatment seen only in MDA-MB-231 cells. To address this issue, we examined another triple negative basal breast cancer cell line, MDA-MB-436. However, the MDA-MB-436 cells were inherently migratory in Matrigel and BMP4 did not induce any additional effects (data not shown). Thus we conclude that the effects of BMP4 cannot be simply explained by the molecular subtype of the cell line. Neither could we link the BMP4-induced phenotypes to other known cell line characteristics, such as the histological type, mutational status, or tumorigenicity [18].

The BMP antagonist Gremlin was able to reverse the MDA-MB-231 stellate phenotype, demonstrating that the effect is truly due to the action of BMP4. Similarly, a broad spectrum MMP inhibitor Batimastat was able to inhibit the BMP4-induced branching of the MDA-MB-231 cells, indicating that the phenomenon required the action of matrix metalloproteinases (MMPs). Unexpectedly, Batimastat also reduced the growth of the cells, both with and without BMP4. MMPs have been shown to cleave intracellular or transmembrane proteins, thereby releasing factors that regulate cell proliferation, apoptosis, invasion and angiogenesis [51-54]. MMP9 has been particularly shown to possess growth-promoting effects [55,56]. Shon et al. [12] found BMP4 to suppress the activity of MMP9 in MDA-MB-231 cells, albeit in 2D culture, but in our 3D experiments the expression level of *MMP9* was too low to allow accurate measurements and thus MMP9 is unlikely to explain the growth suppressive effects of Batimastat. Nevertheless, examination of the expression of *MMP*s targeted by Batimastat revealed upregulation of *MMP3* and *MMP14* in BMP4-treated compared to vehicle-treated cells. Similar induction of *MMP3* or *MMP14* expression was not seen in the non-migratory BT-474 cells, further suggesting a mechanistic link between these *MMPs* and the stellate phenotype in MDA-MB-231 cells. A recent study also showed that BMP4 induces the expression of multiple MMPs, including *MMP3* and *MMP14*, in mouse mammary fibroblasts and it also modestly induces the expression of *MMP3* in cancer associated human mammary fibroblasts and to a greater degree in normal human mammary fibroblasts [57]. In contrast, Otto et al. [58] found BMP4 to inhibit *MMP3* mRNA and protein expression in C3H10T1/2 stem cells, and this inhibition was related to adipogenetic differentiation. These opposing results are likely to reflect cell-type and context-specific differences.

The exact mechanisms behind *MMP3* and *MMP14* induction upon BMP4 treatment in MDA-MB-231 cells remain to be revealed. *MMP3* has in its promoter a binding element for AP-1, which is in turn known to be regulated by BMP4 [59,60], thereby representing a likely link between BMP4 and MMP3. However, previous data from other BMP/TGF-β family members suggest that additional signaling pathways may also contribute to the MMP induction. In MDA-MB-435 melanoma cells, TGF-β-induced upregulation of MMP14 has been shown to be dependent on the ERK1/2, PI3K, and JNK pathways [61] and in MDA-MB-231 cells TGF-β induced the expression of many *MMPs*, including *MMP14*, through the p38 MAP kinase [62]. Similarly, BMP2 has been shown to increase the expression of *MMP9* in gastric cancer cells through AKT, ERK and NF-κB signaling cascades [63]. Taken together, multiple signaling pathways may be involved in the BMP4-induced upregulation of MMP expression.

## Conclusions

In conclusion, the data provided in this study demonstrate that Matrigel provides a more relevant environment to study the effects of biological factors on breast cancer cell behavior than the synthetic PEG gel. The responses of MDA-MB-231 and MDA-MB-361 cells to BMP4 were partly different in 2D than in 3D culture, thus strongly arguing for validation of 2D data in an appropriate 3D environment. Nevertheless, BMP4 retained its bifunctional role of reducing cell proliferation and inducing migration in 3D, albeit not in the same cell line. Finally, this study also delivered further evidence on the molecular mechanisms behind the BMP4-induced phenotypes.

## Abbreviations

2D: Two-dimensional; 3D: Three-dimensional; BMP4: Bone morphogenetic protein 4; MMP: Matrix metalloproteinase; PEG gel: Polyethyleneglycol gel; TGF-β: Transforming growth factor β.

## Competing interests

The authors declare that they have no competing interests.

## Authors’ contributions

MA and RJ conducted the experiments and wrote the manuscript. KJU consulted on the 3D culture experiments and participated in their design. ARM conducted initial experiments and helped in drafting the manuscript. EA and AK conceived of the study, participated in the design and helped to draft the manuscript. All authors approved the final version of the manuscript.

## Pre-publication history

The pre-publication history for this paper can be accessed here:

http://www.biomedcentral.com/1471-2407/13/429/prepub

## Supplementary Material

Additional file 1: Table S1 Gene specific primers and probes. UPL (Universal Probe Library) probes were purchased from Roche. Click here for file

Additional file 2: Figure S1 BMP4 treatment reduces BT-474 cell growth in 2D cell culture. Cells were grown in the presence of 100 ng/ml BMP4 or vehicle and proliferation was measured using the alamarBlue reagent and by counting the cells at indicated time points. Relative proliferation (mean + s.d.) compared to vehicle is shown. *P < 0.05, **P < 0.01. Click here for file

Additional file 3: Figure S2 BMP4 does not influence MDA-MB-361 cells grown in Matrigel but decreases cell proliferation in PEG gels. Cells were grown in Matrigel (a—c) or PEG gel (d—f) supplemented with 100 ng/ml BMP4 or vehicle. Images were taken as indicated in Figure 2 and representative examples from day 14 are shown. Scale bars 200 μm. (b, e) Cell proliferation and (c, f) area covered by cell clusters were measured and are presented as in Figure 2, *P < 0.05, **P < 0.01. Click here for file

Additional file 4: Figure S3 BMP4 influences the expression of cyclin B1, cyclin B2, pCDC2 and p21. The expression levels of a set of known cell cycle regulators were examined using western blotting. MDA-MB-361 and T-47D cells were grown as monolayers and harvested 24 hours after the treatment with 100 ng/ml BMP4 (+) or vehicle (−). Tubulin was used as a loading control and relative expression levels were calculated with ImageJ. Click here for file
