# Supplementary material for: BMP4 inhibits the proliferation of breast cancer cells and induces an MMP-dependent migratory phenotype in MDA-MB-231 cells in 3D environment
Source: BMC Cancer. 2013 Sep 22;13:429. doi: 10.1186/1471-2407-13-429 (PMC3848934; doi:10.1186/1471-2407-13-429)
Supplement: Additional file 1: Table S1 — Gene specific primers and probes. UPL (Universal Probe Library) probes were purchased from Roche. [file 1471-2407-13-429-S1.docx]

**Table S1. Gene specific primers and probes.** UPL (Universal Probe Library) probes were purchased from Roche.

| *MMP* | Forward primer | Reverse primer | UPL probe number |
| --- | --- | --- | --- |
| *MMP1* | CAGAGATGAAGTCCGGTTTTTC | GGGGTATCCGTGTAGCACAT | #26 |
| *MMP2* | TGCTGGAGACAAATTCTGGA | GATGGCATTCCAGGCATC | #60 |
| *MMP3* | CCAGGTGTGGAGTTCCTGAT | CATCTTTTGGCAAATCTGGTG | #72 |
| *MMP7* | GCTGACATCATGATTGGCTTT | TCTCCTCCGAGACCTGTCC | #72 |
| *MMP9* | ATCCGGCACCTCTATGGTC | CTGAGGGGTGGACAGTGG | #43 |
| *MMP14* | GCCTTGGACTGTCAGGAATG | AGGGGTCACTGGAATGCTC | #37 |
| *ADAM17* | TTTGAGACTGCCCAGAAGAAG | GCGGGCACTCACTGCTAT | #79 |
