# Supplementary figures and images for: BMP4 inhibits the proliferation of breast cancer cells and induces an MMP-dependent migratory phenotype in MDA-MB-231 cells in 3D environment
Source: BMC Cancer. 2013 Sep 22;13:429. doi: 10.1186/1471-2407-13-429 (PMC3848934; doi:10.1186/1471-2407-13-429)

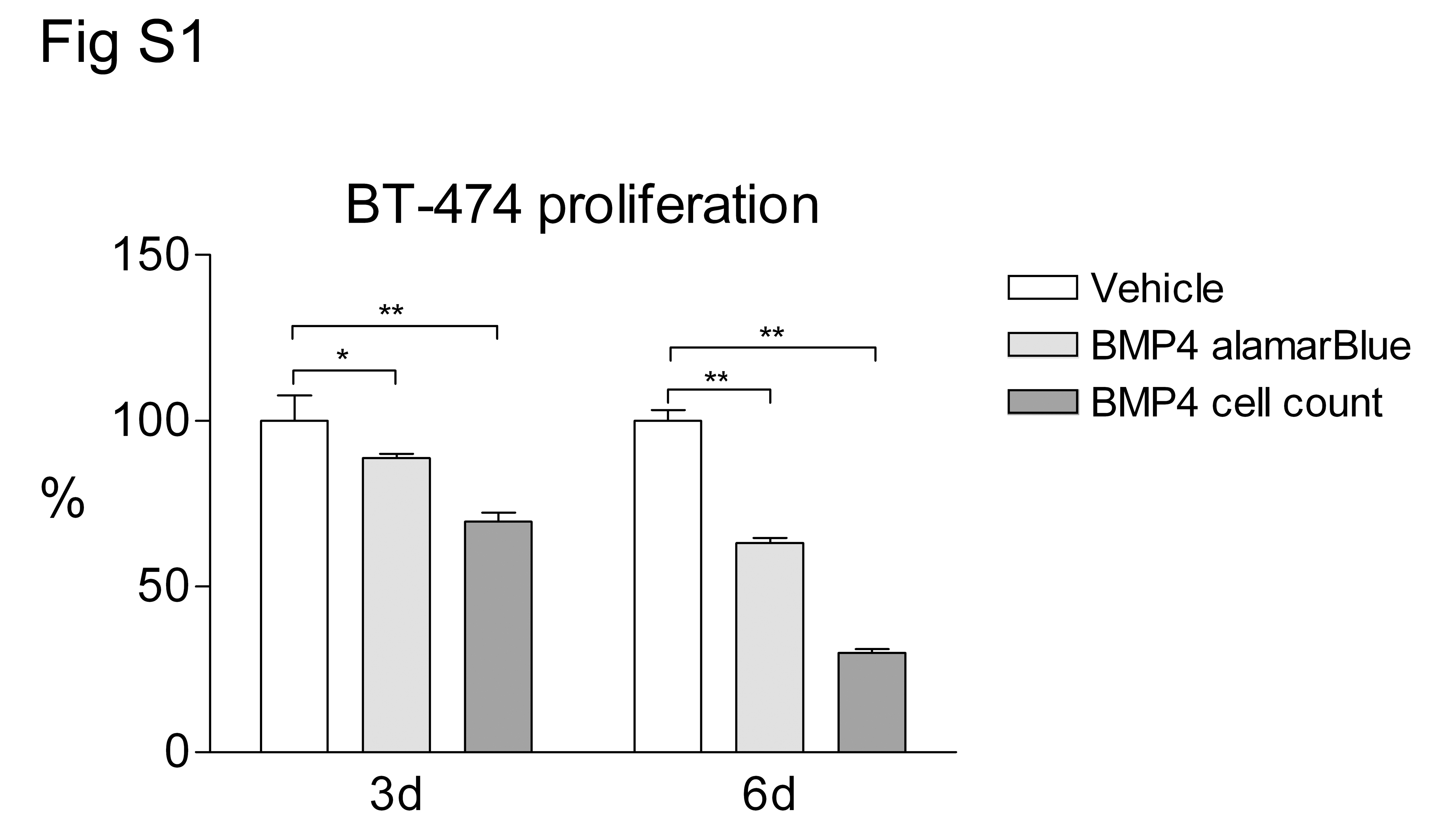

Supplement: Additional file 2: Figure S1 — BMP4 treatment reduces BT-474 cell growth in 2D cell culture. Cells were grown in the presence of 100 ng/ml BMP4 or vehicle and proliferation was measured using the alamarBlue reagent and by counting the cells at indicated time points. Relative proliferation (mean + s.d.) compared to vehicle is shown. *P < 0.05, **P < 0.01. [file 1471-2407-13-429-S2.jpeg]

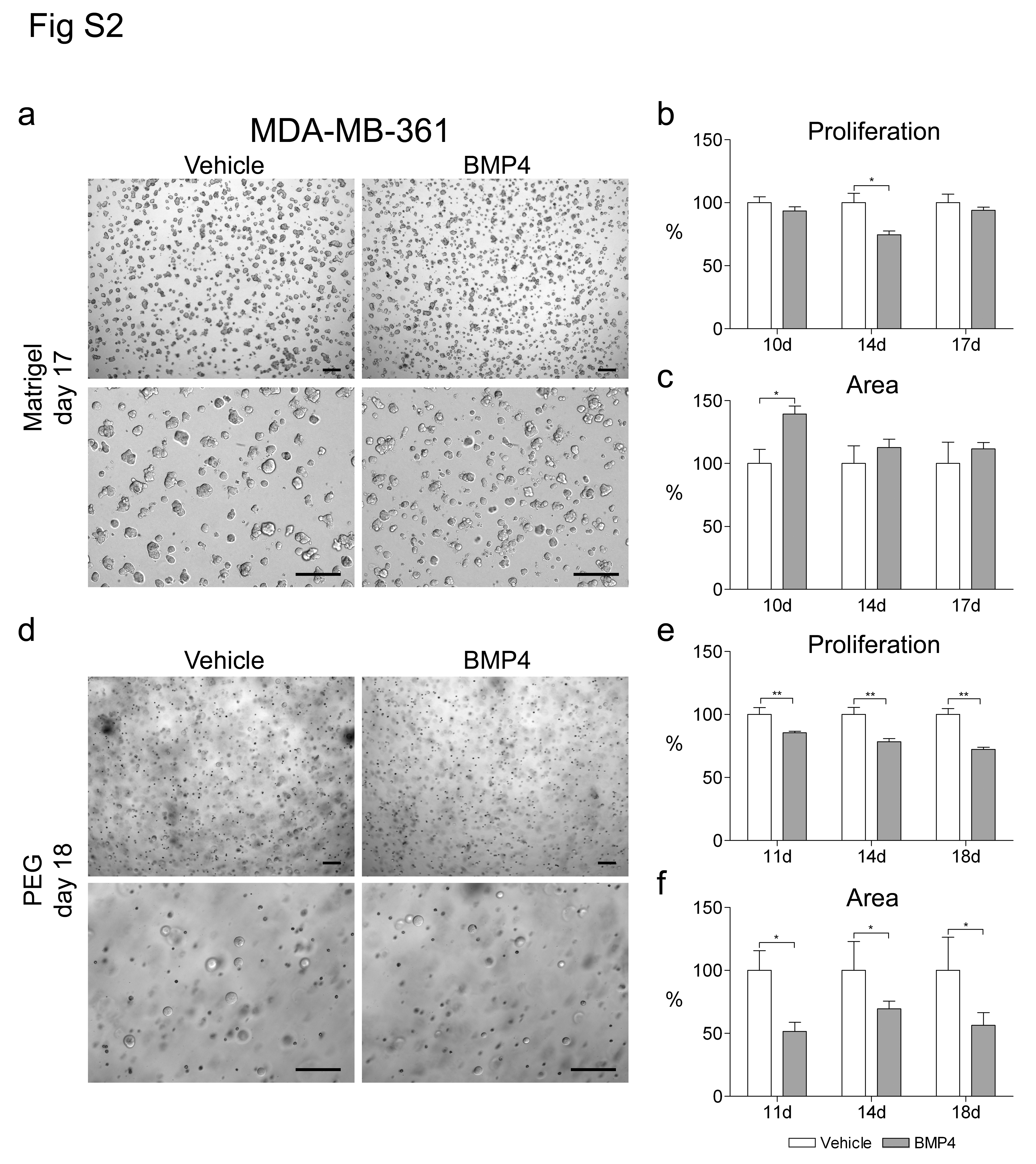

Supplement: Additional file 3: Figure S2 — BMP4 does not influence MDA-MB-361 cells grown in Matrigel but decreases cell proliferation in PEG gels. Cells were grown in Matrigel (a—c) or PEG gel (d—f) supplemented with 100 ng/ml BMP4 or vehicle. Images were taken as indicated in Figure 2 and representative examples from day 14 are shown. Scale bars 200 μm. (b, e) Cell proliferation and (c, f) area covered by cell clusters were measured and are presented as in Figure 2, *P < 0.05, **P < 0.01. [file 1471-2407-13-429-S3.jpeg]

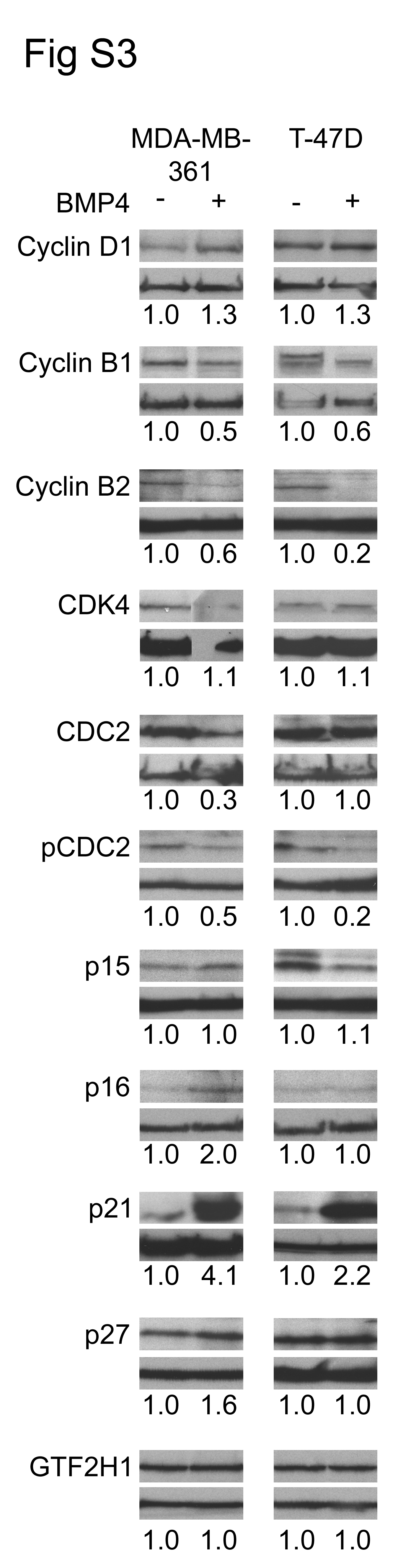

Supplement: Additional file 4: Figure S3 — BMP4 influences the expression of cyclin B1, cyclin B2, pCDC2 and p21. The expression levels of a set of known cell cycle regulators were examined using western blotting. MDA-MB-361 and T-47D cells were grown as monolayers and harvested 24 hours after the treatment with 100 ng/ml BMP4 (+) or vehicle (−). Tubulin was used as a loading control and relative expression levels were calculated with ImageJ. [file 1471-2407-13-429-S4.jpeg]
